# Osteoclast biology in the single-cell era

**DOI:** 10.1186/s41232-022-00213-x

**Published:** 2022-09-02

**Authors:** Masayuki Tsukasaki, Hiroshi Takayanagi

**Affiliations:** Department of Immunology, Graduate School of Medicine and Faculty of Medicine, The University of Tokyo, 7-3-1, Hongo, Bunkyo-ku, Tokyo, 113-0033 Japan

**Keywords:** Osteoclast, Single-cell analysis, Osteoblast, Bone metabolism, Osteoimmunology

## Abstract

Osteoclasts, the only cells that can resorb bone, play a central role in bone homeostasis as well as bone damage under pathological conditions such as osteoporosis, arthritis, periodontitis, and bone metastasis. Recent studies using single-cell technologies have uncovered the regulatory mechanisms underlying osteoclastogenesis at unprecedented resolution and shed light on the possibility that there is heterogeneity in the origin, function, and fate of osteoclast-lineage cells. Here, we discuss the current advances and emerging concepts in osteoclast biology.

## Background

Since C. H. Robin [1] first depicted a multinucleated cell on the bone surface, early studies using the latest technologies at the time such as electron microscopy, bone marrow chimera techniques, and in vitro osteoclast culture systems have been employed to establish the concept that osteoclasts are hematopoietic-origin cells that exclusively possess a bone-resorbing capacity [2]. Takahashi et al. provided the first direct evidence that osteoblastic cells support osteoclast formation in an in vitro co-culture system, suggesting that osteoblastic cells may produce a certain molecule(s) capable of inducing osteoclastogenesis [3]. Receptor activator of NF-κB ligand (RANKL) was then identified as the long-sought osteoclast differentiation factor expressed by osteoblastic cells [4–7]. RANKL is indispensable for osteoclast differentiation in humans and mice; the loss or mutation of RANKL or its receptor RANK causes osteopetrosis due to a complete lack of osteoclasts in both species [8–10]. To date, there is no clear evidence of RANKL-independent osteoclastogenesis or any factor that is able to replace RANKL functions [10–12]. Macrophage colony-stimulating factor (M-CSF) is also an essential osteoclastogenic molecule, the lack of which causes an osteopetrotic phenotype [13, 14]. Osteoblastic cells, including osteoblasts and osteocytes, express both RANKL and M-CSF to support osteoclastogenesis in vitro [10].

**Fig. 1:**
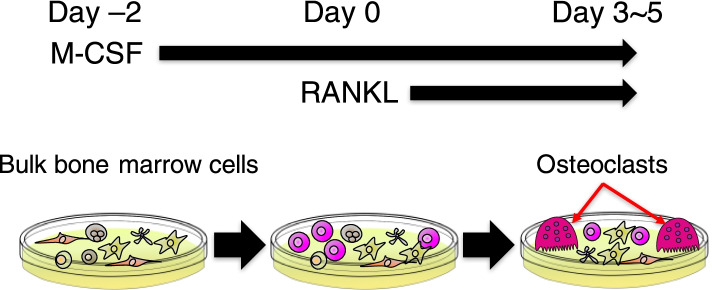
Schematic of the osteoclast culture system. Murine bulk bone marrow cells are treated with M-CSF for 2 days, and then, these cells are stimulated with RANKL in the presence of M-CSF. Osteoclasts appear in the culture system after 3–5 days of RANKL stimulation. The osteoclast culture contains heterogeneous populations of cells, only a portion of which is able to differentiate into mature osteoclasts

Recombinant RANKL and M-CSF turned out to be sufficient to induce osteoclastogenesis in murine bone marrow cells, and this type of in vitro osteoclast culture system has been widely used in the bone research field (Fig. 1) [4]. Transcriptome studies using the in vitro osteoclast culture as well as investigations of the naturally occurring and genetically modified osteopetrotic animals revealed a number of essential molecules for osteoclast differentiation and activation, providing fundamental insights into osteoclast biology [15]. Furthermore, recent studies using single-cell RNA sequencing (scRNA-seq) have further advanced our understanding of the regulatory mechanisms underlying osteoclastogenesis. In this review, we will summarize basic knowledge and discuss recent progress and emerging questions in the osteoclast biology field.

## The RANKL signaling pathway in osteoclastogenesis

After RANKL was discovered as the genuine master regulator of osteoclast differentiation, osteoclast researches centered on the elucidation of downstream signaling pathways of the RANKL/RANK axis. Large-scale screening (e.g., microarrays and bulk RNA-seq) using the in vitro osteoclast culture identified various RANKL-inducible genes and shed light on the intracellular signaling networks involved in osteoclastogenesis (Fig. 1).

**Fig. 2:**
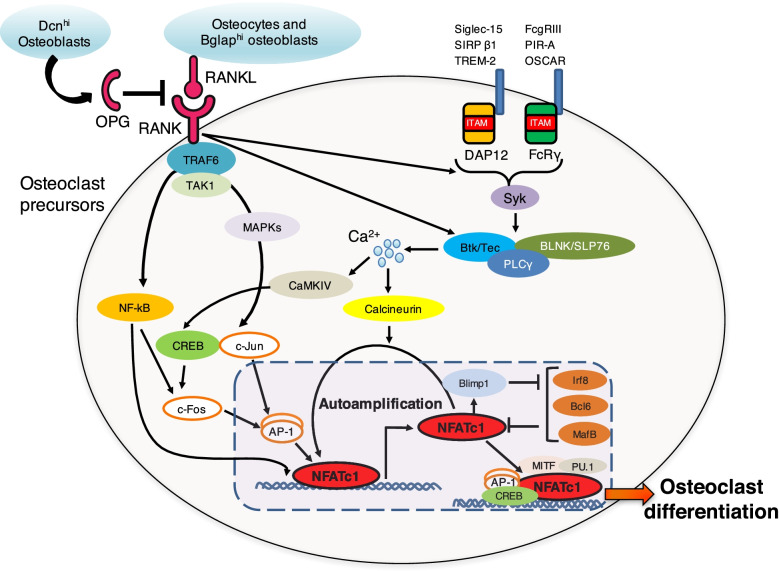
Molecular mechanisms underlying osteoclast differentiation. RANKL, the master regulator of osteoclastogenesis, is expressed by osteocytes and Bglap^hi^ osteoblasts. Dcn^hi^ osteoblasts locally produce OPG to inhibit osteoclast differentiation and activation. RANKL binding to RANK expressed by osteoclast progenitors results in the activation of signaling cascades including MAPK and NF-κB pathways via TRAF6 and TAK1. The RANKL/RANK signal cooperates with signaling from ITAM-containing immunoglobulin-like receptors such as TREM-2, SIRP ββ, Siglec-15, OSCAR, PIR-A, and FcγRIII. These signaling cascades ultimately lead to the auto-amplification of NFATc1, the master transcription factor of osteoclastogenesis

RANKL binding to the RANK expressed by osteoclast progenitors results in the activation of signaling cascades, including the mitogen-activated protein kinase (MAPK) and NF-κB pathways via the adaptor protein tumor necrosis factor receptor-associated factor 6 (TRAF6) and the kinase TGF-β-activated kinase-1 (TAK1) [15, 16]. The activation of the MAPK and NF-κB pathways facilitates the formation of the c-Fos and c-Jun complex, the AP-1 dimer critical for osteoclast differentiation [15, 16]. The RANKL/RANK signal cooperates with signaling from its co-stimulatory receptors: immunoreceptor tyrosine-based activation motif (ITAM)-containing immunoglobulin-like receptors such as triggering receptor expressed on myeloid cells 2 (TREM-2), signal-regulatory protein β-1 (SIRP ββ), sialic acid-binding immunoglobulin-like lectin 15 (Siglec-15), osteoclast-associated receptor (OSCAR), paired immunoglobulin-like receptor A (PIR-A), and FcγRIII [15, 16]. These receptors are associated with ITAM-containing adaptors such as DNAX-activating protein of 12 kDa (DAP12) and the Fc receptor γ-chain (FcRγ) [15, 16]. ITAM phosphorylation leads to the recruitment of spleen tyrosine kinase (Syk), resulting in the activation of adaptor proteins such as B-cell linker (BLNK) and SH2 domain-containing leukocyte protein of 76 kDa (SLP76), which function as scaffolds that recruit the Tec kinases Btk/Tec and phospholipase Cγ (PLCγ) [15, 16]. This complex stimulates the activation of calcium signaling, leading to the auto-amplification of nuclear factor of activated T cells c1 (NFATc1), the master transcription factor of osteoclastogenesis [15, 16]. The inhibition of the expression of anti-osteoclastogenic transcription factors (e.g., (Irf8), Bcl6, and MafB) by NFATc1 is also required for osteoclast differentiation [15, 16] (Fig. 2). NFATc1 choreographs the expression of osteoclastogenic genes including DC-STAMP, a transmembrane protein essential for osteoclast fusion [17, 18]. Although certain factors (e.g., DC-STAMP [17], OC-STAMP [19], ATP6v0d2 [20], and dynamin [21]) required for osteoclast fusion have been reported, precise molecular mechanisms underlying how these fusogenic factors cooperatively facilitate osteoclast fusion remain largely unclear [22].

Intriguingly, the signaling molecules important for osteoclastogenesis turned out to be the factors that had been identified and studied in the field of immunology [15, 16]. The NFAT transcription factors play a role in T-cell development and activation, while immunoglobulin-like receptors are important for the activation of innate immune cells. Tec family tyrosine kinases are critical for B-cell maturation and immunoglobulin production. The osteoclast signal studies have highlighted the shared molecules and mechanisms between the bone and immune systems, thereby critically contributing to the establishment of the concept of “osteoimmunology” [15, 16].

## Where does OPG come from?

Osteoprotegerin (OPG) is a circulating decoy receptor for RANKL, functioning as an essential negative regulator of osteoclastogenesis by inhibiting the interaction between RANKL and its receptor RANK [23–26]. Since OPG was first cloned as a “osteoprotective” factor in 1997, it has long been thought that the OPG level in the bone tissue is an important determinant of bone mass, and that the serum OPG level may also be associated with bone pathologies such as osteoporosis and rheumatoid arthritis. However, the cellular source of OPG in vivo has long been unknown, as OPG is expressed in various tissues and circulates in the blood. Furthermore, it has been obscure whether OPG functions only at the site of production or circulates to other tissues so as to function in an endocrine manner.

Recently, two independent studies generated OPG-floxed mice and demonstrated that the OPG locally produced by osteoblasts, but not circulating OPG, is essential for bone homeostasis [27, 28]. Deletion of OPG in osteoblasts by using *Sp7*-Cre or *Dmp1*-Cre markedly decreased bone volume, whereas OPG deletion in B cells (*Mb1*-Cre) or osteocytes (*Sost*-Cre) did not [27, 28]. Intriguingly, osteoblast-specific OPG-deficient mice retained normal serum OPG levels, indicating that circulating OPG does not affect bone metabolism [27, 28]. This was also true in the thymus and intestine, two other organs where the RANKL/RANK/OPG system plays a key role [27]. Medullary thymic epithelial cell (mTEC) and intestinal microfold cell (M cell) were shown to be the primary sources of OPG in the thymus and intestine, respectively [27]. Deletion of locally produced OPG disrupted thymic and intestinal homeostasis without affecting the serum OPG level [27]. These findings highlight the importance of the tight regulation of RANKL activity by local OPG production in vertebrate homeostasis.

Intriguingly, an analysis of the bone tissue scRNA-seq dataset showed that OPG is highly expressed in the osteoblast subtype characterized by a high expression of an extracellular matrix protein decorin (Dcn) [27]. RANKL mRNA expression was detected in the other osteoblast subtype (Bglap^hi^ osteoblastic cells), but not in the Dcn^hi^ osteoblastic cells [27]. Since osteoblastic cells control osteoclastogenesis by producing both RANKL and OPG in the in vitro co-culture system, it has long been assumed that the osteoblastic cells that produce RANKL and OPG are the same population [29]. However, it is unknown whether all the osteoblastic cells equally produce OPG, or there is an osteoblastic cell subset that highly produces OPG. The data obtained from scRNA-seq analysis suggests that RANKL-expressing and OPG-expressing cells may represent distinct subsets [27]. Further studies are needed to understand the heterogeneity and functional diversity of osteoblasts (Fig. 2).

## Single-cell landscape of osteoclastogenesis

**Fig. 3:**
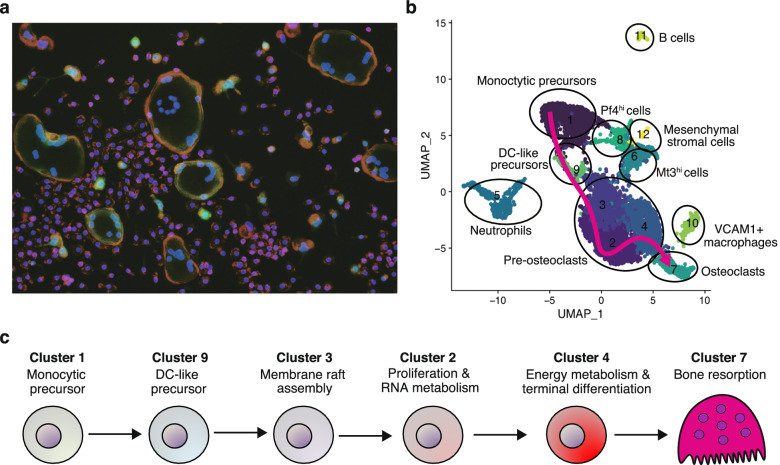
Single-cell landscape of osteoclastogenesis. **a** Representative image of osteoclast differentiation culture system after 3 days of RANKL stimulation in bone marrow cells from CtsK-Cre CAG-CAT-EGFP mouse. The multinucleated giant cells labeled with EGFP are osteoclasts. Most of the cells in the culture system failed to differentiate into mature osteoclasts. Green (EGFP), CTSK; red, actin; blue, DAPI. **b** The osteoclast differentiation trajectory estimated by pseudotime analysis using the scRNA-seq data obtained from the in vitro osteoclast culture system. **c** Schematic of the stepwise cell fate decision pathways during osteoclastogenesis unveiled by scRNA-seq

The understanding of the molecular mechanisms underlying osteoclastogenesis has largely relied on the data obtained from transcriptome analyses, including microarrays and bulk RNA-seq performed on the in vitro osteoclast culture, and a number of the important signaling molecules for osteoclastogenesis have been identified using this approach (Fig. 1). However, this osteoclast formation system has a critical limitation; the culture system contains heterogeneous populations of cells (Figs. 1 and 3a). This cellular heterogeneity has hampered a precise understanding of the molecular mechanisms underlying osteoclastogenesis and made it difficult to identify key osteoclastic genes that are expressed at low levels in osteoclasts or expressed by contaminating cells [30].

A recent study that applied scRNA-seq on the osteoclast culture system identified the stepwise cell fate decision pathways during osteoclast differentiation [30] (Fig. 3b and c). Unexpectedly, in silico trajectory analysis suggested that monocytic precursor cells transitioned through CD11c-expressing dendritic cell (DC)-like status in the early stage of osteoclastogenesis [30]. Several studies have proposed that DCs may function as osteoclast precursor cells based on the findings that FACS-sorted CD11c^+^ cells can differentiate into osteoclasts in in vitro culture and in vivo transfer models [31–34]. However, it was reported that osteoclast formation is not decreased in mice lacking mature DCs, suggesting that DCs are dispensable for osteoclastogenesis under physiological conditions [35]. Thus, the contribution of DCs to osteoclast formation has been controversial. The in silico trajectory inference based on the scRNA-seq data suggested that CD11c is transiently expressed in early stage osteoclast precursors [30]. This computational prediction was validated by demonstrating that *CD11c*-Cre-mediated deletion of RANK significantly inhibited osteoclast formation in vivo and in vitro [30]. The transient expression of CD11c in osteoclast precursors may resolve the controversy over the role of DCs in osteoclast formation. It will be interesting to investigate the physiological relevance of the transient CD11c expression in osteoclastogenesis.

DC-like precursors then undergo stepwise biological processes, membrane raft assembly, proliferation, cell-cycle arrest, and the terminal differentiation into mature osteoclasts [30] (Fig. 3b and c). Cited2 was identified as a transcriptional regulator, the expression of which was progressively elevated during the trajectory of osteoclast differentiation [30]. Cited2-deficient cells only give rise to proliferating pre-osteoclasts and fail to proceed to cell cycle-arrested pre-osteoclasts, suggesting that Cited2 is required for cell-cycle arrest, an essential step in the terminal differentiation of osteoclasts [30]. This hypothesis is consistent with previous findings that Cited2 and its binding partner CBP/p300 are crucial for cell cycle arrest in various cell types [36, 37].

Interestingly, in silico pseudotime analysis suggested that there is another trajectory in the osteoclast culture system: the monocytic precursors differentiate into “failed osteoclasts” that express certain osteoclast markers such as tartrate-resistant acid phosphatase (TRAP), matrix metalloproteinase 9 (MMP9), and cathepsin K (CtsK), but do not have sufficient potential to become bona fide osteoclasts [30]. It remains unclear why the same precursor cells have a different fate under the identical culture conditions and whether such “failed osteoclasts” actually exist and are functionally effective in vivo.

There are several studies that have applied scRNA-seq to the primary cells collected from bone tissues, but osteoclast-lineage cells are not always covered by the scRNA-seq datasets, probably due to limitations of cell size and the strong bone-adhesive nature of these cells [38–41]. A study performing scRNA-seq on tdTomato^+^ cells sorted from the bone marrow of Col2-Cre Rosa-tdTomato mice captured not only mesenchymal cells but also hematopoietic contaminants, including osteoclast-lineage cells [42]. Trajectory analysis suggested that monocytic precursors underwent bilineage differentiation into mature osteoclasts with a progressive increase in Cited2 expression and also into another macrophage cluster (termed Mϕβ) which exhibits low-level expression of certain osteoclast markers such as TRAP, MMP9, and CtsK [42]. These data are similar to the scRNA-seq data obtained from in vitro osteoclastogenesis [30], and it will be interesting to determine whether the Mϕβ cells in vivo represent the “failed osteoclasts” found in the in vitro culture system.

## Heterogeneity in the origin, function, and fate of osteoclasts

Single-cell analysis is a powerful strategy for the deconvolution of heterogeneous populations of cells, and the development and widespread use of scRNA-seq technology have led to the discovery of novel subsets within cell types previously believed to be comprised of a single population. Thus, questions have arisen about whether there is heterogeneity in osteoclast-lineage cells (Table 1). Traditionally, the heterogeneity of osteoclasts has been discussed in terms of anatomical localization and the types of hard tissue resorbed. It is reported that calvarial osteoclasts have a larger size than long bone osteoclasts and may utilize different proteases to degrade the bone matrix [2, 43, 44]. For instance, MMP2-deficient mice exhibit increased bone volume only in the calvariae and not in the long bones [45]. These findings suggest that the osteoclasts localized to intramembranous and endochondral bones may have distinct characteristics.

**Table 1:** The heterogeneity of hard tissue-resorbing cells. Diversity in hard tissue-resorbing cells at different sites and biological settings

Cell types	Characteristics	References
Calvarial osteoclasts	Larger in size and utilize distinct proteases from long bone osteoclasts	[43–45]
Odontoclasts	Resorb dental tissues, but differences from osteoclasts are not clear	[46]
Vascular-associated osteoclasts (VAOs)	Closely associated with type H vessels to regulate blood vessel growth	[47]
Septoclasts	Cartilage-resorbing mesenchymal cells characterized by expression of FABP5 and MMPs	[48]
Type H endothelial cells	Produce MMP9 to degrade cartilage	[47]
Arthritis-associated osteoclastogenic macrophages (AtoMs)	Arthritis-associated osteoclast precursors controlled by transcription factor FoxM1	[49]
Osteoclast precursors with myeloid suppressor function	Expand in the bone marrow of arthritic mice and inhibit T-cell proliferation	[50]
Osteoclasts associated with bone loss induced by colitis and estrogen deficiency	Containing heterogeneous population with distinct immune regulatory functions	[51, 52]
Fracture-associated osteoclasts	Derived from yolk-sac macrophage descendants residing in the adult spleen	[53]
Fracture-associated circulating CX3CR1+ precursors	Migrate to the fracture sites and differentiate into osteoclasts	[54]
Obesity-associated osteoclast precursors	High-fat diet-induced monocytic MDSCs capable of differentiating into osteoclasts	[55]
Osteomorphs	Daughter cells produced by osteoclast fission capable of fusing back into osteoclasts	[56]

As subpopulations of osteoclasts, odontoclasts, which resorb teeth, and chondroclasts, which are involved in cartilage resorption, have been documented by histological studies (Table 1). Odontoclasts express osteoclast markers and exhibit structural features similar to osteoclasts, but the detailed characteristics of odontoclasts remain thus far only poorly understood [46]. Since RANKL-deficient mice completely lack osteoclasts but display only minor growth plate cartilage abnormalities, it has long been enigmatic which cell types are responsible for the function of “chondroclasts” [57]. Recently, specialized endothelial cells (type H) [47] and mesenchymal-derived septoclasts [48] were shown to produce MMPs, critically contributing to cartilage resorption. Unlike osteoclast of which differentiation is controlled by RANKL provided by osteoblastic cells, septoclast specification was regulated by the Notch ligand delta-like 4 provided by endothelial cell.

Intriguingly, a detailed analysis of the osteoclasts localized at the bone/cartilage interface suggested that they display low CtsK expression levels and are closely associated with type H endothelial cells [47]. These cells were termed vascular-associated osteoclasts (VAOs) and shown to contribute to blood vessel growth, but not cartilage degradation [47]. Thus, cartilage degradation might be mediated by cell types distinct from osteoclasts.

Osteoclasts are essential not only for physiological bone remodeling but also for pathological bone destruction [58, 59], and the concept of “disease-associated osteoclasts” is currently attracting attention in the field (Table 1). Hasegawa et al. identified a novel arthritis-associated osteoclast precursor macrophage (AtoMs) which gives rise to pathogenic osteoclasts in the arthritic synovium in a manner dependent on the transcription factor FoxM1 [49]. During arthritis, CD11b^–/lo^ Ly6C^hi^ cells are expanded in the bone marrow, and these cells may function as osteoclast precursors as well as myeloid-derived suppressor cells (MDSCs) inhibiting T-cell proliferation [50]. In other bone loss models induced by colitis and estrogen deficiency, certain myeloid cell populations were also reported to be capable of differentiating into osteoclasts and regulating T cells in vitro, but their pathological relevance in vivo remains unclear [51, 52]. In a high-fat diet-induced obesity model, monocytic MDSCs were shown to be expanded and to have the capacity to differentiate into osteoclasts in vitro [55] (Table 1). As “disorder-specific monocyte/macrophage subtypes” corresponding to certain diseases have been reported [60], it is possible that osteoclasts are composed of multiple subsets corresponding to a variety of disorders. Investigations into such disease-associated osteoclasts may contribute to the development of therapeutic strategies against pathological bone damage without affecting physiological bone metabolism.

**Fig. 4:**
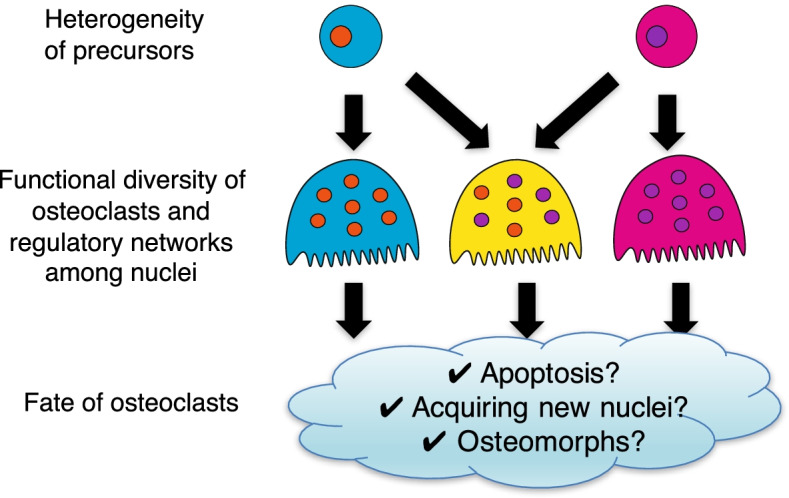
Emerging mysteries in the osteoclast biology. Single-cell studies have provoked new questions in the osteoclast biology field. Osteoclast precursors may comprise different subsets depending on life stages and pathologies; however, functional difference among osteoclasts derived from distinct precursors remains unclear. Given that osteoclast precursors can fuse one another to differentiate into osteoclasts, it will be important to elucidate the regulatory mechanisms of the multinuclear system to understand the functional diversity of osteoclasts. Although osteoclasts are thought to die quickly by apoptosis after resorbing bone, novel hypotheses regarding the fate of osteoclasts have emerged. Further studies are needed to draw a comprehensive picture of osteoclast life cycle and its functional diversity in health and disease

The origin of osteoclasts has been shown to vary depending on the life stage [61]. During development and the early life stages, osteoclasts originate from yolk-sac erythromyeloid progenitors (EMPs), whereas bone marrow hematopoietic stem cells (HSCs) are the source of osteoclasts in adults [61]. During fracture healing, CX3CR1^+^ yolk-sac macrophage descendants residing in the adult spleen migrate to the bone injury sites and differentiate into osteoclasts [53]. Another paper has also shown that osteoclasts at the fracture sites are derived from circulating CX3CR1^+^ cells, supporting the notion that the fracture-associated osteoclasts are supplied by the bloodstream [54] (Table 1). Intriguingly, it was shown that HSC-derived and EMP-derived precursors can fuse with each other to differentiate into osteoclast [53]. Thus, in order to understand the functional diversity of osteoclasts derived from distinct precursors, it will be important to elucidate the regulatory mechanisms of the multinuclear system, i.e., the interactions (or hierarchy) among the nuclei (Fig. 4).

There may be heterogeneity in the fate of osteoclasts. It has long been thought that osteoclasts have a life span of 2–4 weeks and die quickly by apoptosis after the termination of bone resorption [62]. However, a recent study proposed that osteoclasts may live more than 6 months by acquiring new nuclei from circulating precursors based on the following observation; when parabionts of Csf1r-Cre: Rosa26LSL-YFP mouse (osteoclast precursors labeled with YFP) and Csf1r-Cre: Rosa26LSL-tdTomato mouse (osteoclast precursors labeled with tdTomato) were separated after 4 weeks of shared blood circulation, most osteoclasts retained the expression of both YFP and tdTomato, even at 6 months after the separation [61]. Since this observation cannot rule out the possibility that it is not osteoclasts but rather “osteoclast precursors” that are long-lived, as proposed by previous reports [63–65], further studies are needed to clarify the life span of mature osteoclasts in vivo. Recently, a study with intravital imaging approaches showed that high-dose RANKL injection led to an alternative cell fate in which osteoclasts fission into daughter cells, termed “osteomorphs” (Table 1). Osteomorphs can fuse and recycle back into osteoclasts, and scRNA-seq analysis suggested that osteomorphs are transcriptionally distinct from osteoclasts and macrophages [56]. Given that high-dose RANKL injection is required to observe osteoclast fission, osteomorphs may develop under pathological rather than physiological conditions. Further studies are required to clarify the relevance of osteoclast fission and osteomorphs in health and disease.

## Concluding remarks and perspectives

Single-cell technology has brought about a new era in life science, and the pathophysiology of the skeletal system is now being described by the data gathered from individual cells. Recent studies using scRNA-seq analysis have unveiled the molecular mechanisms underlying osteoclastogenesis at an unprecedented level of resolution. Furthermore, increasing attention has been paid to the heterogeneity that characterizes the origin, function, and fate of osteoclasts, but we are still far from understanding the comprehensive picture (Fig. 4). Where do osteoclasts come from, what do they do, and where do they go? Further investigations into osteoclast biology using single-cell technology will provide key insights that will undoubtedly contribute to advances in therapeutic strategies targeting osteoclasts in skeletal diseases.

## Data Availability

Not applicable.
